# Effect of ω-3 polyunsaturated fatty acid-supplemented parenteral nutrition on inflammatory and immune function in postoperative patients with gastrointestinal malignancy: A meta-analysis of randomized control trials in China

**DOI:** 10.1097/MD.0000000000010472

**Published:** 2018-04-20

**Authors:** Yajie Zhao, Chengfeng Wang

**Affiliations:** Department of Pancreatic and Gastric surgery, National Cancer Center/Cancer Hospital, Chinese Academy of Medical Sciences and Peking Union Medical College, Beijing, China.

**Keywords:** gastrointestinal malignancy, meta-analysis, omega-3 polyunsaturated fatty acid, parenteral nutrition

## Abstract

**Background:**

: There are no consensus regarding the efficacy of omega-3polyunsaturated fatty acids (PUFAs) on inflammatory and immune function in postoperative patients with gastrointestinal malignancy.

**Methods:**

The literatures published randomized control trials (RCT) were searched in PubMed, Embase, Scopus, Cochrane Library, CNKI, Weipu, and Wanfang Databases. The immune efficacy outcomes of ω-3 polyunsaturated fatty acid-supplemented parenteral nutrition in patients with gastrointestinal malignancy were compared.

**Results:**

Sixteen RCTs involving 1008 patients (506 in the omega-3 group, 502 in the control group) were enrolled into the analysis. The results of meta-analysis: the cell immunity: The proportions of CD3^+^, CD4^+^, CD4^+^/CD8^+^ in the omega-3 group were significantly higher than those in the control group (CD3^+^: WMD = 4.48; 95% CI, 3.34–5.62; *P* < .00001; *I*^2^ = 0%; CD4^+^: WMD = 5.55; 95% CI, 4.75–6.34; *P* < .00001; *I*^2^ = 0%; CD4^+^/CD8^+^: WMD = .28; 95% CI, 0.13–0.44; *P* = .0004; *I*^2^ = 81%). In the humoral immunity: The levels of IgA, IgM and IgG in the omega-3 group were significantly higher than those in the control group (IgA: WMD = 0.31; 95% CI, 0.25–0.37; *P* < .00001; *I*^2^ = 0%; IgM: WMD = 0.12; 95% CI, 0.06–1.81; *P* < .00001; *I*^2^ = 0%; IgG: WMD = 1.19; 95% CI, 0.80–1.58; *P* < .00001; *I*^2^ = 0%). The count of lymphocyte in the omega-3 group was significantly higher than that in the control group (WMD = 0.22; 95% CI, 0.12–0.33; *P* < .0001; *I*^2^ = 40%). In the postoperative inflammatory cytokine: The levels of interleukin-6, tumor necrosis factor (TNF)-α and C-reactive protein in the omega-3 group were significantly lower than those in the control group (IL-6: WMD = −3.09; 95% CI, −3.91 to 2.27; *P* < .00001; *I*^2^ = 45%; TNF-α: WMD = −1.65; 95% CI, −2.05 to 1.25; *P* < .00001; *I*^2^ = 28%; CRP: WMD = −4.28; 95% CI, −5.26 to 3.30; *P* < .00001; *I*^2^ = 37%). The rate of postoperative infective complications in the omega-3 group was significantly lower than that in the control group (OR = 0.36; 95% CI, 0.20–0.66; *P* = .0008; *I*^2^ = 0%).

**Conclusion:**

This meta-kanalysis confirmed that early intervention with Omega -3 fatty acid emulsion in gastrointestinal cancer can not only improve the postoperative indicators of immune function, reduce inflammatory reaction, and improve the postoperative curative effect but also improve the immune suppression induced by conventional PN or tumor. Therefore, postoperative patients with gastrointestinal cancer should add omega-3 unsaturated fatty acids in their PN formula. Further high-quality RCTs are needed to verify its efficacy.

## 1 Introduction

Fat emulsions are important dietary supplements in parenteral nutrition (PN) that can provide essential fatty acids and energy and maintain cell structure and human adipose tissue. Essential fatty acids for the human body are the omega-6 group linoleic acid and omega-3 group alpha linolenic acid, which are both polyunsaturated fatty acids (PUFA). PUFA can be divided into 4 families—omega-3, omega-6, omega-7, and omega-9. Among them, the omega-3 family mainly includes alpha linolenic acid, 20 carbon five acid (EPA), and 22 carbon six acid (DHA). Omega-3 fish oil fatty milk is extracted from deep sea fish oil, the main components of which are EPA and DHA. In recent years, studies have shown that omega-3 PUFA can increase the stability of the omega cell membrane, regulate immune function, block excessive inflammatory reaction,^[1]^ reduce the occurrence of systemic inflammatory response syndrome (SIRS) and multiple organ dysfunction syndrome (MODS) and infectious complications, and inhibit tumor growth.

Traditional fat emulsion, such as long chain fatty emulsion (LCT), is primarily sourced from soybean oil, and its main component is omega-6 fatty acid. Arachidonic acid, an important derivative of linoleic acid, synthesizes PGI_2_, LTB_4_, and TXA_2_ by a series of enzymatic reactions. These substances are important proinflammatory mediators in inflammatory response and inhibit immune function. Omega-3 fish oil fat emulsion is effective in improving cellular immune function and inhibiting inflammatory response. The possible mechanism is that the EPA and DHA in omega-3 fish oil fat emulsion are further metabolized to PGE_3_, PGI_3_, TXA_2_, and LTB_5_, which can competitively inhibit the release and metabolism of arachidonic acids (AA), thereby reducing the body's inflammatory response and protecting from immune-mediated damage. In addition, EPA and DHA can also change the lipid composition and cell function of T-lymphocyte membrane, increase cell membrane stability, and affect the cellular immune function.^[2,3]^

Some studies have investigated the efficacy of omega-3 PUFA-enriched nutrition for patients with gastrointestinal malignancy undergoing surgery, and the primary results indicated that the immunological function of omega-3 PUFAs would be helpful in preventing postoperative infectious complications.^[4]^ However, the results and conclusions of these studies were not entirely consistent owing to limited sample size, different study designs, and potential bias. Therefore, we performed a meta-analysis of all relevant randomized control trials (RCTs) with the main focus on the efficacy of omega-3 PUFAs on inflammatory and immune function in postoperative patients with gastrointestinal malignancy.

## 2 Materials and methods

### 2.1 Literature-search strategy

**Figure 1:**
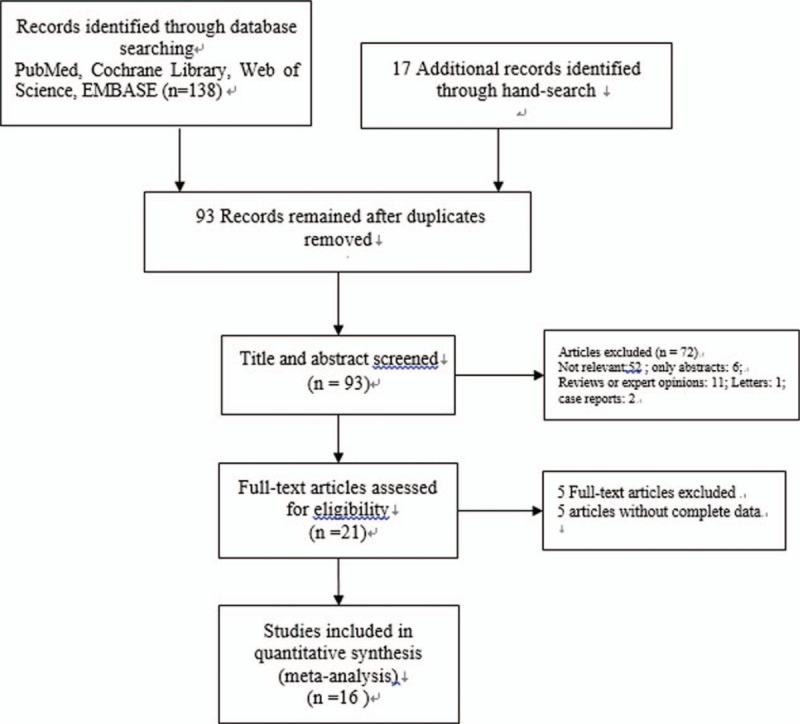
PRISMA flow diagram depicting the selection process.

We searched for journal articles published from January 2000 to June 2017 both electronically and manually. We searched the databases of PubMed, the Cochrane Library, Web of Science, EMBASE, and the Chinese Biomedicine Database for the following search terms: (“omega-3” OR “n-3” OR “polyunsaturated” OR “FO” OR “omega-3 fatty acid” OR “n-3 polyunsaturated fatty acid”) AND (“fatty acid” OR “fish oil”) AND (“cancer” OR “carcinoma” OR “tumor” OR “neoplasms”) AND (“surgery” OR “operation”) AND (“colorectal” OR “colon” OR “rectum” OR “gastrointestinal” OR “gastric”). Both MeSH words and free terms were included in the search. No language restriction was applied and the search was performed by 2 independent researchers. Final inclusion of articles was determined by consensus; when this failed, a third author adjudicated. The results of the search strategy are shown in Fig. 1. All analyses were based on previous published studies; thus, no ethical approval and patient consent are required.

### 2.2 Inclusion criteria

Studies that met the following criteria were included: studies that evaluated the inflammatory and immune function of omega-3 PUFA-enriched nutrition for patients undergoing surgery for gastrointestinal malignancy. The omega-3 PUFAs should have been administered as an adjuvant in the study group (omega-3 group), and the duration of administration had to be short-term postsurgery; the study was the most recent publication in the case of multiple publications; and RCTs that had access to the full-text.

### 2.3 Exclusion criteria

The following studies were excluded: those wherein details of PN were not available; those with no comparison of omega-3 group and control group; those in which the study outcomes did not include complete or available postoperative data; those which reported data used in a later study; and case reports, abstracts, letters, comments, and reviews without original data, and studies that presented insufficient data.

### 2.4 Data extraction

The following detailed data were independently extracted by 2 investigators and checked by the other authors: title; authors; year of publication; country; study design; number of patients; interventions (daily dose, duration of omega-3 PUFA administration, and type of PN); postoperative serum inflammatory cytokines (C-reactive protein [CRP], tumor necrosis factor [TNF-α], and interleukin-6 [IL-6]); postoperative humoral immune function markers such as immunoglobulin (IgA, IgM, and IgG) levels; cellular immune function CD3^+^, CD4^+^, CD8^+^ cell counts and CD4^+^:CD8^+^ ratio; lymphocyte count; and the incidence of postoperative infectious complications.

### 2.5 Statistical analysis

Meta-analysis was conducted with Review Manager (version 5.3.0) software. Odds ratios (ORs) used to analysis the categorical variables and 95% confidence interval (CI) values were reported. Weighted mean difference (WMD) used to analysis the continuous variables and 95% CI values were reported. The Mantel–Haenszel, Chi-square, and *I*^2^ tests were used to test the heterogeneity between studies. *I*^2^ > 50%, this suggested significant heterogeneity, a random effects model was applied. If *I*^2^ < 50%, this suggested not significant heterogeneity, a fixed effects model was applied. If *P* < .05, this considered statistically significant. Funnel plots were used to evaluate potential publication bias.

### 2.6 Characteristics of the included studies and quality assessment

In this meta-analysis, 16 randomized clinical trials RCTs were included. The total number of patients was 1008, of whom 506 was omega-3 group and 502 was control group. The characteristics of all the included studies are shown in Table 1. The RCTs were qualitatively analyzed using modified Jadad scale system, which was used to assess randomization, concealment of allocation, blinding, and withdrawals in the study. Each item was given a score of 0 to 2 and 7 score in total. If the total score was ≥4, the RCT was of high quality (Table 2).

**Table 1:**
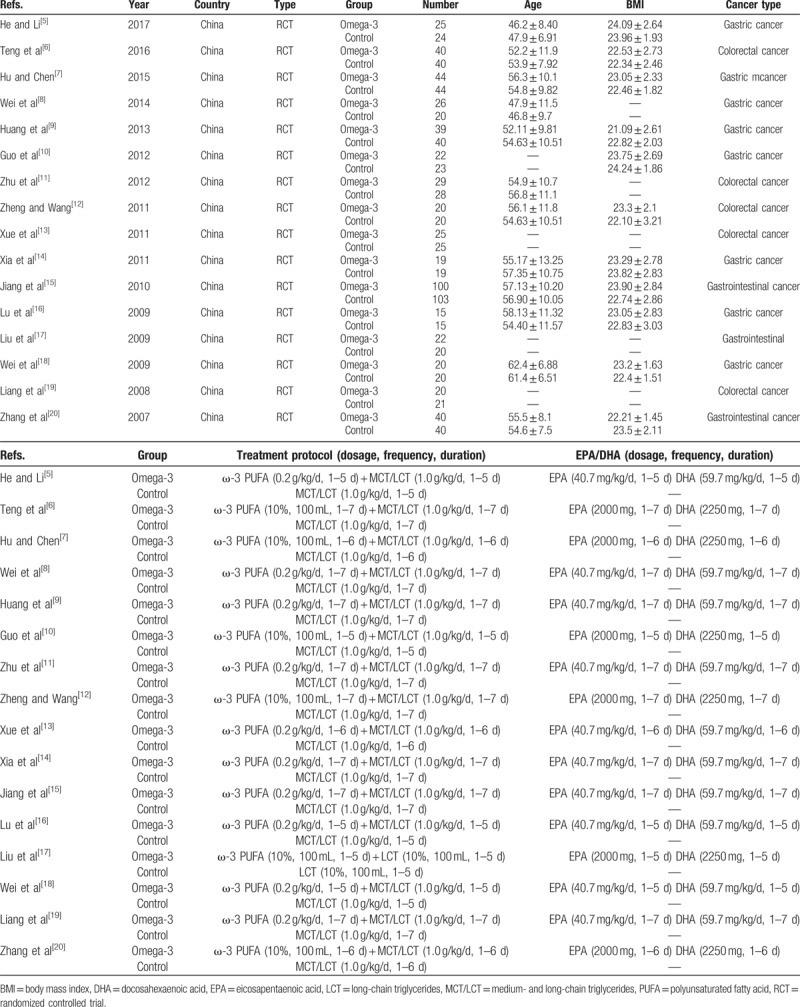
The characteristics of all the included studies.

**Table 2:**
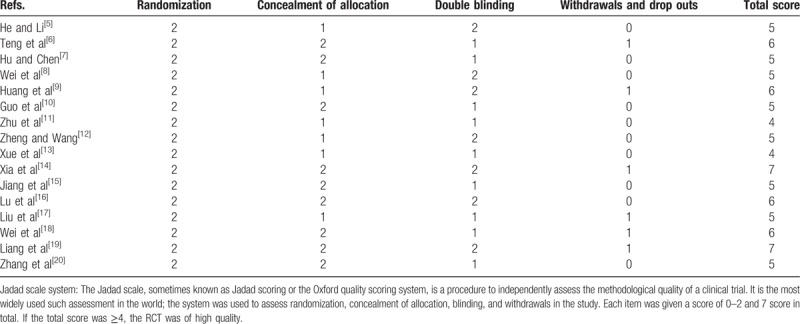
Jadad scale system for randomized controlled trials.

### 2.7 Assessment of the risk of bias of RCTs

**Figure 2:**
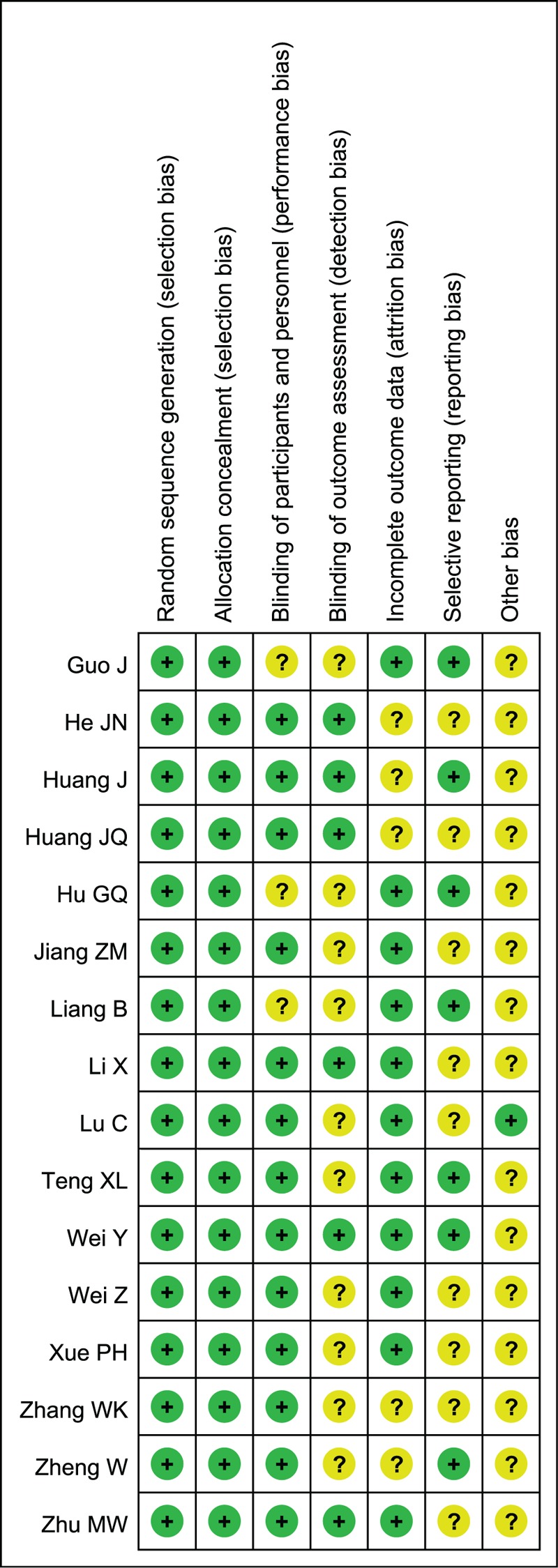
Risk of bias of RCTs: Assessment was based on a quality checklist recommended in the Cochrane Handbook. “+” indicated a “low” risk of bias; “?” an “uncertain” risk of bias.

For the included RCTs, assessment of the bias risk involved 6 parameters: sequence generation, allocation concealment, blinding, incomplete outcome data, selective reporting bias, and other potential sources of bias (Fig. 2).

## 3 Meta-analysis results

### 3.1 Summary statistics of meta-analyses

The statistical findings of meta-analyses comparing postoperative outcomes of ω-3 group and control group are summarized in Table 3.

**Table 3:**
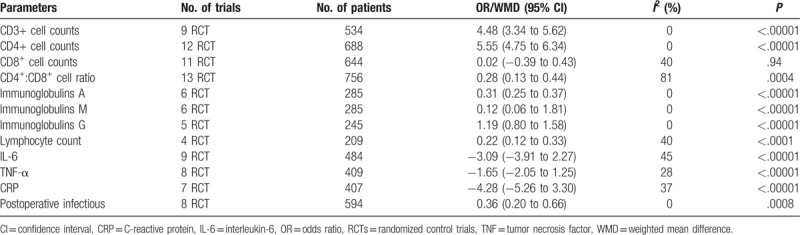
Summary statistics of meta-analyses comparing outcomes of omega-3 group and control group.

### 3.2 Postoperative cellular immune function

#### 3.2.1 Postoperative CD3^+^ cell counts

**Figure 3:**
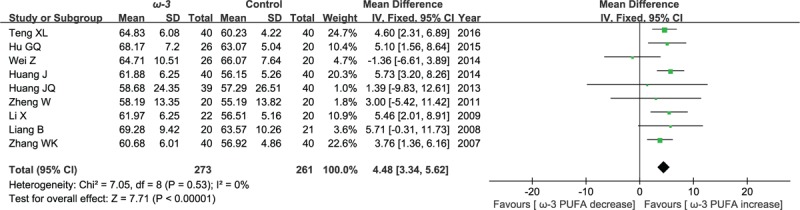
Meta-analysis of the postoperative CD3^+^ cell counts (%).

Nine included studies reported the effect of ω-3 polyunsaturated fatty acid-supplemented on postoperative CD3^+^ cell counts (%). The results of meta-analysis show that the value of postoperative CD3^+^ cell counts increased in response to ω-3 PUFA supplementation (WMD = 4.48; 95% CI, 3.34–5.62; *P* < .00001; *I*^2^ = 0%). Therefore, using a fixed model (Fig. 3).

#### 3.2.2 Postoperative CD4^+^ cell counts

**Figure 4:**
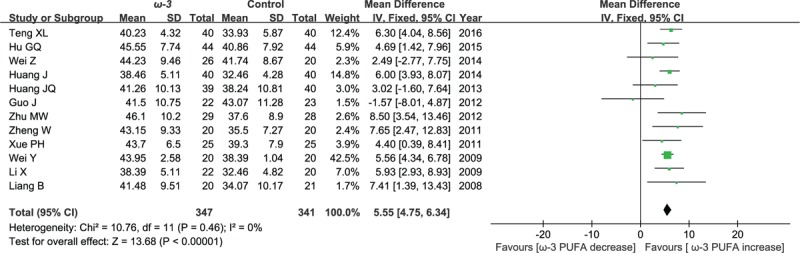
Meta-analysis of the postoperative CD4^+^ cell counts (%).

Twelve included studies reported the effect of ω-3 polyunsaturated fatty acid-supplemented on postoperative CD4^+^ cell counts (%), we pooled data from the 12 studies to comparing ω-3 group with control group. The results of meta-analysis show that the value of postoperative CD4+ cell counts increased in response to ω-3 PUFA supplementation (WMD = 5.55; 95% CI, 4.75–6.34; *P* < .00001; *I*^2^ = 0%). Therefore, using a fixed model (Fig. 4).

#### 3.2.3 Postoperative CD8^+^ cell counts

**Figure 5:**
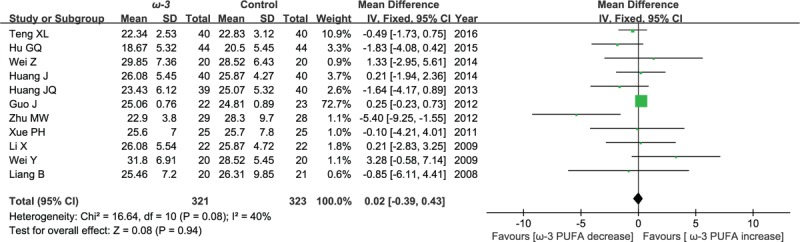
Meta-analysis of the postoperative CD8^+^ cell counts (%).

Eleven included studies reported the effect of ω-3 polyunsaturated fatty acid-supplemented on postoperative CD8^+^ cell counts (%), we pooled data from the 11 studies to comparing omega-3 group with control group. The results of meta-analysis indicate that there is no significant difference between the 2 groups in terms of postoperative CD8^+^ cell counts (WMD = 0.02; 95% CI, −0.39–0.43; *P* = .94; *I*^2^ = 40%). Therefore, using a fixed model (Fig. 5).

#### 3.2.4 Postoperative CD4^+^:CD8^+^ cell ratio

**Figure 6:**
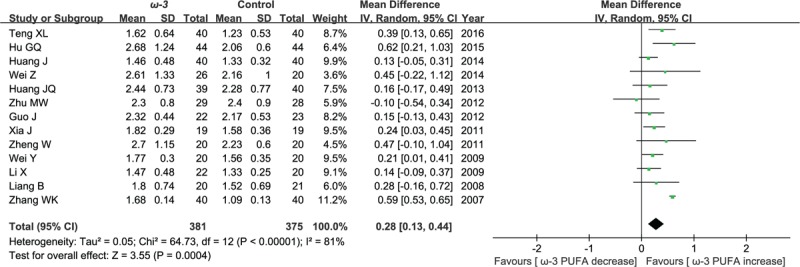
Meta-analysis of the postoperative CD4^+^:CD8^+^ cell ratio.

Thirteen included studies reported the effect of ω-3 polyunsaturated fatty acid-supplemented on postoperative CD4^+^:CD8^+^ cell ratio. The results of meta-analysis show that the value of postoperative CD4^+^:CD8^+^ cell ratio increased in response to ω-3 PUFA supplementation (WMD = 0.28; 95% CI, 0.13–0.44; *P* = .0004; *I*^2^ = 81%). Therefore, using a random model (Fig. 6).

### 3.3 Postoperative humoral immune function

#### 3.3.1 Postoperative immunoglobulins A

**Figure 7:**
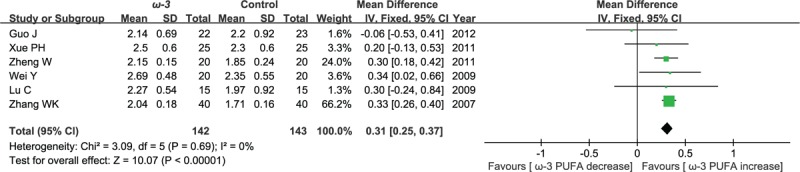
Meta-analysis of the postoperative immunoglobulins A (g/L).

Six included studies reported the effect of ω-3 polyunsaturated fatty acid-supplemented on postoperative immunoglobulins A (g/L). We pooled data from the 6 studies to comparing ω-3 group with control group. The results of meta-analysis show that the value of postoperative immunoglobulins A increased in response to ω-3 PUFA supplementation (WMD = 0.31; 95% CI, 0.25–0.37; *P* < .00001; *I*^2^ = 0%). Therefore, using a fixed model (Fig. 7).

#### 3.3.2 Postoperative immunoglobulins M

**Figure 8:**
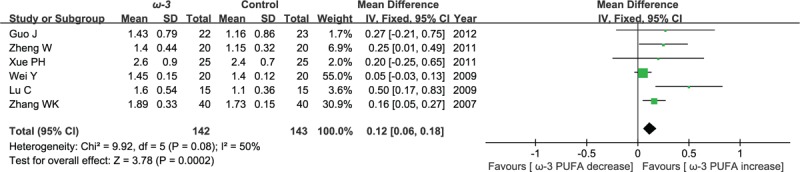
Meta-analysis of the postoperative immunoglobulins M (g/L).

Six included studies reported the effect of ω-3 polyunsaturated fatty acid-supplemented on postoperative immunoglobulins M (g/L). The results of meta-analysis show that the value of postoperative immunoglobulins M increased in response to ω-3 PUFA supplementation (WMD = 0.12; 95% CI, 0.06–0.18; *P* = .0002; *I*^2^ = 50%). Therefore, using a fixed model (Fig. 8).

#### 3.3.3 Postoperative immunoglobulins G

**Figure 9:**
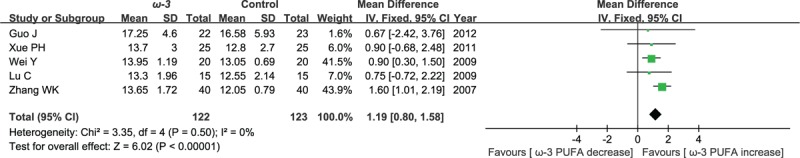
Meta-analysis of the postoperative immunoglobulins G (g/L).

Five included studies reported the effect of ω-3 polyunsaturated fatty acid-supplemented on postoperative immunoglobulins G (g/L). The results of meta-analysis show that the value of postoperative immunoglobulins G increased in response to ω-3 PUFA supplementation (WMD = 1.19; 95% CI, 0.80–1.58; *P* < .00001; *I*^2^ = 0%). Therefore, using a fixed model (Fig. 9).

### 3.4 Postoperative lymphocyte count

**Figure 10:**
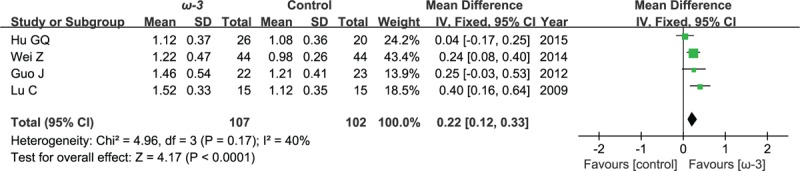
Meta-analysis of the postoperative lymphocyte count (10^9^/L).

Four included studies reported the effect of ω-3 polyunsaturated fatty acid-supplemented on postoperative lymphocyte count (10^9^/L). The results of meta-analysis show that the value of postoperative lymphocyte count increased in response to ω-3 PUFA supplementation (WMD = 0.22; 95% CI, 0.12–0.33; *P* < .0001; *I*^2^ = 40%). Therefore, using a fixed model (Fig. 10).

### 3.5 Postoperative inflammatory cytokine

#### 3.5.1 Postoperative values of IL-6

**Figure 11:**
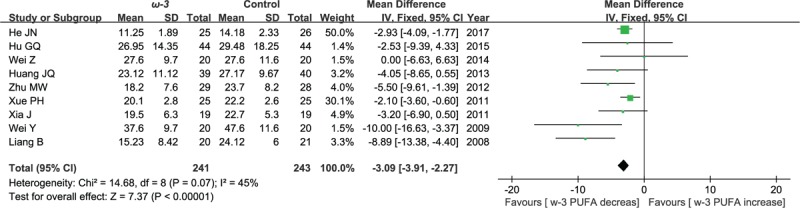
Meta-analysis of the postoperative values of IL-6 (ng/L).

Nine included studies reported the effect of ω-3 polyunsaturated fatty acid-supplemented on postoperative values of IL-6 (ng/L). The results of meta-analysis show that postoperative values of IL-6 decreased in response to ω-3 PUFA supplementation (WMD = −3.09; 95% CI, −3.91–2.27; *P* < .00001; *I*^2^ = 45%). Therefore, using a fixed model (Fig. 11).

#### 3.5.2 Postoperative values of TNF-α

**Figure 12:**
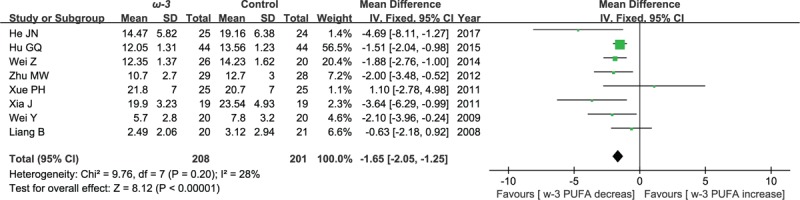
Meta-analysis of the postoperative values of TNF-α (ng/L).

Eight included studies reported the effect of ω-3 polyunsaturated fatty acid-supplemented on postoperative values of TNF-α (ng/L). The results of meta-analysis show that postoperative values of TNF-α decreased in response to ω-3 PUFA supplementation (WMD = −1.65; 95% CI, −2.05–1.25; *P* < .00001; *I*^2^ = 28%). Therefore, using a fixed model (Fig. 12).

#### 3.5.3 Postoperative values of CRP

**Figure 13:**
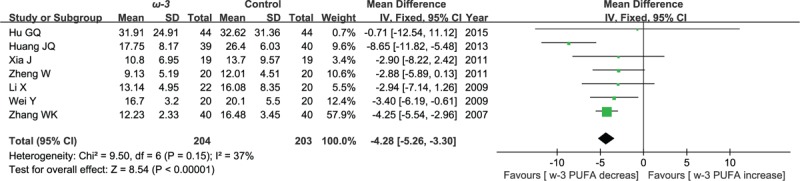
Meta-analysis of the postoperative values of CRP (mg/L).

Seven included studies reported the effect of ω-3 polyunsaturated fatty acid-supplemented on postoperative values of CRP (mg/L). The results of meta-analysis show that postoperative values of CRP decreased in response to ω-3 PUFA supplementation (WMD = −4.28; 95% CI, −5.26 to 3.30; *P* < .00001; *I*^2^ = 37%). Therefore, using a fixed model (Fig. 13).

### 3.6 Postoperative incidence of infectious

**Figure 14:**
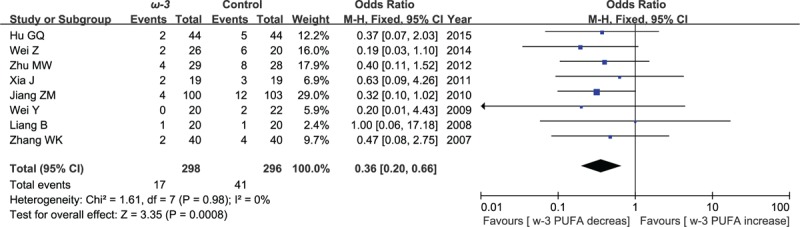
Meta-analysis of the postoperative incidence of infectious.

Eight included studies reported the effect of ω-3 polyunsaturated fatty acid-supplemented on postoperative incidence of infectious. The results of meta-analysis showed that postoperative incidence of infectious decreased in response to ω-3 PUFA supplementation (OR = 0.36; 95% CI, 0.20–0.66; *P* = .0008; *I*^2^ = 0%). Therefore, using a fixed model (Fig. 14).

#### 3.6.1 Publication bias

**Figure 15:**
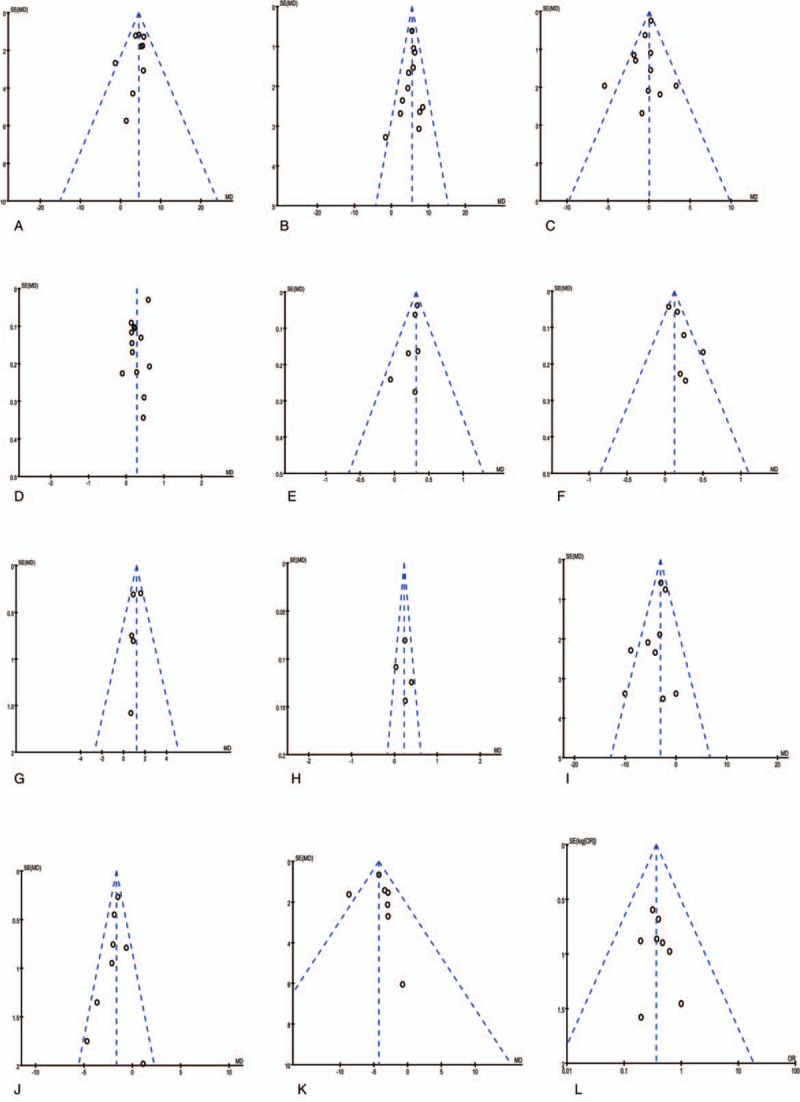
Funnel plots: Funnel plots were created to assess the publication bias in our meta-analysis of included studies. In the absence of publication bias, it assumes that studies with high precision will be plotted near the average, and studies with low precision will be spread evenly on both sides of the average, creating a roughly funnel-shaped distribution. (A) Postoperative CD3^+^ cell counts, (B) postoperative CD4^+^ cell counts, (C) postoperative CD8^+^ cell counts, (D) postoperative CD4^+^:CD8^+^ cell ratio, (E) postoperative immunoglobulins A, (F) postoperative immunoglobulins M, (G) postoperative immunoglobulins G, (H) postoperative lymphocyte count, (I) postoperative values of IL-6, (J) postoperative values of TNF-α, (K) postoperative values of CRP, (L) postoperative incidence of infectious.

Deviation from this shape can indicate publication bias. There was no evident asymmetry in the funnel plots (Fig. 15), suggesting a low probability of publication bias.

## 4 Discussion

Most patients with gastrointestinal cancer have varying degrees of malnutrition and low immune function because of poor appetite, digestion, and absorption dysfunction, and tumor consume and produce some immunosuppressive factors.^[21–23]^ This not only affects the healing and prognosis of anastomotic stoma but also worsens the patients’ prognoses; and the risk of postoperative secondary infection and various complications understandably increases. Studies have shown that changes in immune function play an important role in host tumor recurrence or metastasis, and low immune function can cause accelerated diffusion of tumor cells. Therefore, it is of great clinical significance to improve the nutritional status and the immune function of such patients.^[1,24–27]^

T lymphocyte-mediated cell-mediated immunity plays an important role in anti-tumor immune response.^[28–31]^ The ratio of T lymphocyte subsets CD3^+^, CD4^+^, CD8^+^, and CD4^+^/CD8^+^ is a sensitive index to reflect the cellular immune function of an organism. The level of CD3^+^ T cells reflects the overall level of cellular immunity. CD4^+^ T cells can promote B-cell differentiation (to induce antibody production), activate other cells so that they secrete lymphatic factors, and play a mediating role in inflammatory reactions. CD8^+^ T cells are a type of immune suppression cell, as they inhibit antibody secretion and T-cell proliferation, CD8^+^ cells may also represent cytotoxic cells. Some studies have shown that the ratio of CD3^+^, CD4^+^, and CD4^+^/CD8^+^ decreased and the ratio of CD8^+^ increased in the peripheral blood of patients in the first postoperative week, which was suggestive of inhibited cellular immune function. The ratio change due to surgical trauma and the body in high metabolic stress postsurgery resulted in inhibited T lymphocyte function, thereby reducing the number of cells and strength of the immune response. The results of our meta-analysis showed that the value of CD3^+^, CD4^+^, and CD4^+^/CD8^+^ ratio increased in ω-3 groups on the sixth postoperative day. The ratio of CD3^+^, CD4^+^ T lymphocyte subsets in ω-3 group was significantly higher than the control group, suggesting that the ω-3 unsaturated fatty acid can enhance postoperative cellular immunity of patients with gastrointestinal malignancy. Meanwhile, an increased ratio of CD4^+^/CD8^+^ can enhance cellular immunity, promote cell activation and differentiation, and enhance humoral immunity. This meta-analysis suggests that these indicators of cellular immune function of patients in the ω-3 group was better than that of the control group. Immunoglobulins are a group of proteins that act as antibodies and are mainly present in human blood, tissue fluids, and exocrine fluids. Immunoglobulins are an important index to examine the humoral immune function of an organism. This meta-analysis showed that there was a clear difference between the ω-3 and control groups in terms of IgA, IgG, and IgM levels on the sixth postoperative day. The results indicated that ω-3 PUFAs can improve indicators of cellular and humoral immune function.

TNF-α, IL-6, and CRP play an important role in response to tissue injury and inflammation in early trauma.^[32,33]^ TNF-α and IL-6 are pre-inflammatory factors that have an important role in induction and regulation of inflammatory response. TNF-α is a protein produced by LPS-stimulated monocyte macrophage, which is a promoter of multidirectional inflammation and inflammation.^[34]^ TNF-α can activate neutrophils, macrophages, and other inflammatory cells, and can induce IL-6 secretion by vascular endothelial cells. IL-6 is an important index to reflect the severity of inflammation and tissue damage. The results of our meta-analysis showed that there was a significant difference between the ω-3 groups and control groups with regard to TNF-α and IL-6 levels on the sixth postoperative day. The values of IL-6 and TNF-α were significantly lower in the ω-3 PUFAs group than the control group, thereby confirming that ω-3 PUFAs can significantly reduce the release of IL-6 and TNF-α in serum and reduce damage to the immune system and enhance immune function. CRP is an acute phase protein synthesized by IL-6-induction of hepatocytes. CRP levels show a rapid and sensitive change in acute trauma and infection and can reflect the change of inflammatory reaction in an organism. Continuous monitoring of CRP after operation is a sensitive index to determine the degree of postoperative stress response and infectious complications and is thus clinically significant. Studies have shown that the majority of patients showed an increase in CRP levels 4 to 12 hours after operation, which reached a peak at 24 to 72 hours, and returned to baseline 14 days after surgery. The results of our meta-analysis showed that the levels of CRP were significantly different between the 2 groups on the sixth postoperative day, with the control group showing significantly higher levels than the ω-3 group. This indicated that ω-3 PUFAs can reduce the postsurgical inflammatory reaction in patients with gastrointestinal tumors.

Peripheral blood neutrophils, eosinophils, basophils, lymphocytes, and monocytes are important components of the body's defense system. In this study, lymphocyte count was significantly higher in ω-3 groups than the control groups. Omega-3 PUFAs improve the body's defense system by the proliferation of lymphocytes, and the meta-analysis result also confirmed that the incidence of infectious complications in ω-3 groups was significantly lower than the control group. Omega-3 PUFAs can regulate the release of inflammatory mediators and promote lymphocyte proliferation to improve the body's defense system, and improve postoperative outcomes in patients. Furthermore, Wei et al reported that ω-3 PUFAs can reduce the expression and level of tumor-related factors such as vascular endothelial growth factor and insulin-like growth factor-1, suggesting that ω-3 PUFAs had inhibitory effects on tumor growth. As all studies did not report the long-term survival of patients, it is necessary to further investigate the long-term clinical prognosis of patients with gastrointestinal cancer after the use of ω-3 PUFAs.

A major limitation of our meta-analysis is that all the included RCTs were Chinese studies. Therefore, the conclusion may not be generalizable to other ethnic populations. Another potential limitation is that experience and methods of perioperative management used at different hospitals and specialist centers could have produced different outcomes and increased the heterogeneity among the included studies.

## 5 Conclusions

Omega-3 fatty acids play an important role in the regulation of inflammatory factors and immune function and improve the nutritional status through a variety of mechanisms. The result of this meta-analysis confirmed that early intervention with ω-3 fatty acid emulsion in gastrointestinal cancer cannot only improve the postoperative indicators of immune function, reduce inflammatory reaction, and improve the postoperative curative effect but also improve the immune suppression induced by conventional PN or tumor. Therefore, postoperative patients with gastrointestinal cancer should add ω-3 unsaturated fatty acids in their PN formula. Further high-quality RCTs are needed to verify its efficacy.

## Author contributions

**Writing – original draft:** Yajie Zhao.

**Writing – review and editing:** Chengfeng Wang.
